# Assessment of the potential of novel Californian grapevine *Trichoderma* isolates to reduce colonization of fungal trunk canker pathogens and *Xylella fastidiosa*

**DOI:** 10.3389/fpls.2025.1609693

**Published:** 2025-09-09

**Authors:** Christopher M. Wallis, Ranjeet Shinde, Margaret L. Ellis, Zachary Gorman, Nalong Mekdara

**Affiliations:** 1 Crop Diseases, Pests and Genetics Research Unit, San Joaquin Valley Agricultural Sciences Center, U.S. Department of Agriculture-Agricultural Research Service, Parlier, CA, United States; 2 Oak Ridge Institute for Science and Engineering, U.S. Department of Energy, Parlier, CA, United States; 3 Department of Plant Science, California State University, Fresno, CA, United States; 4 Chemistry Research Unit, Center for Medical, Agricultural and Veterinary Entomology, U.S. Department of Agriculture-Agricultural Research Service, Gainesville, FL, United States

**Keywords:** biological control agents, Bot canker, dieback, fungal trunk disease, Pierce’s disease

## Abstract

**Introduction:**

Grapevine fungal trunk diseases are cosmopolitan and act to reduce vineyard yields over time. Additionally, Pierce’s disease, caused by *Xylella fastidiosa*, is a fatal disease of grapevines and a major threat wherever it is endemic. These grapevine diseases are generally managed via cultural practices and chemical applications. However, management can be costly due to labor costs or are becoming less effective due to pathogen resistance to pesticides. Thus, there is increasing interest in biological control agents to manage grapevine diseases. Therefore, novel isolates of *Trichoderma* species were collected from grapevine tissues in California with the intention that these would be likely to survive and thrive in the semi-arid and very hot climate present throughout much of the state.

**Methods:**

Genetic analyses and morphology were utilized to identify Californian vineyard-acquired isolates to species or species complex, which yielded several different species: two isolates of *Trichoderma harzianum*, two isolates of *Trichoderma capillare*, and two putative novel *Trichoderma* species. These were examined for activity against fungal trunk pathogens *Diplodia seriata, Eutypa lata*, and *Neofusicoccum parvum* via co-plating and spent media assays. Follow-up greenhouse studies also assessed the ability of isolates to limit fungal pathogen canker development and *Xylella fastidiosa* success over six months. Lastly, field studies tested the ability to limit or remove fungal trunk pathogen colonization of pruned spurs by the *Trichoderma* isolates from this study and two isolates from another study, which were an isolate of *Trichoderma asperellum* and a member of the *Trichoderma saturnisporopsis* species complex.

**Results and Discussion:**

Results potentially yielded *Trichoderma* isolates with some ability to limit fungal pathogens in culture, greenhouse plants, and pruned spurs in the field, and with the ability to be re-isolated after a full field season. However, these isolates were not able to consistently limit *Xylella fastidiosa* titers or Pierce’s disease symptoms. Taken together, these experiments demonstrated the ability of California *Trichoderma* isolates to be deployed as locally sourced biological control agents to protect Californian vineyards as well as those in similar climates.

## 1 Introduction

Grapevines encounter a variety of serious trunk diseases, including those that are rarely fatal but result in decreased yields over time, such as those collectively known as fungal trunk diseases, and those that cause fatal blocking of xylem vessels, namely, Pierce’s disease caused by the bacterium *Xylella fastidiosa* (Sipiora and Cuellar, 2014; Kaplan et al., 2016; Rapicavoli et al., 2018; Baumgartner et al., 2019). The former set of diseases includes *Botryosphaeria* diebacks (caused by pathogens such as *Diplodia seriata* and *Neofusicoccum parvum*), *Eutypa* dieback (caused by *Eutypa lata*), and esca disease (caused by fungi such as *Phaeomoniella chlamydospora*) (Mugnai et al., 1999; Urbez-Torres, 2011; Trotel-Aziz et al., 2019; Martinez-Diaz et al., 2021; Ye et al., 2021). These fungal diseases are spread via conidia during wet weather in the springtime, often due to open pruning wounds within vineyards, as well as through other vineyard cultural practices. Initial introductions generally occur via infected nursery stock. Incidence can quickly build toward 100% in vineyards (approximately by year 7 after planting), and severity can gradually reduce vine productivity until the potential need for replanting the entire vineyard to maintain profitability becomes required (Baumgartner et al., 2019). Currently, there is no cure for fungal trunk diseases, with management generally focused on attempts to reduce spread via delayed or double pruning, better sanitation, occasional applications of pruning wound protectants, and synthetic fungicides (Spagnolo et al., 2017; Travadon et al., 2022). These methods are becoming increasingly costly, as pruning or sanitation practices incur large labor costs, and synthetic pesticides are observed to be becoming less effective due to potential pathogen resistance and are being phased out in many jurisdictions (Urbez-Torres and Gubler, 2009; Brown et al., 2020; Travadon et al., 2022).

As for Pierce’s disease, it is caused by the bacterium *X. fastidiosa* ssp. *fastidiosa* and is spread by the vectoring sharpshooter insects, such as the glassy-winged sharpshooter (*Homalodisca vitripennis*) (Hopkins and Purcell, 2002; Rapicavoli et al., 2018). Pierce’s disease is often fatal, as infections by *X. fastidiosa* affect water transportation in vines, leading to desiccation and death (Sicard et al., 2018). Current management consists of vector control generally by synthetic insecticides, but these are becoming limited in use due to an increasing regulatory environment and building insect resistance (Andreason et al., 2018).

Ultimately, long-term, sustainable management of these diseases would involve improved grapevine materials with natural resistance. Indeed, new cultivars have been released to express a tolerant phenotype to *X. fastidiosa* infection and, as a result, do not develop Pierce’s disease, albeit these do not cover all grape marketing classes (Krivanek et al., 2006).

Until the development of improved cultivars, another possible management option could be the use of biological control agents. Perhaps the most studied and utilized biological control agents are those from the fungal genus *Trichoderma*, which are widely used in agriculture (Woo et al., 2014; Verma et al., 2017). These work generally by direct predation or active antagonization of other fungi, as well as the indirect inhibition of other microbial growth (Harman et al., 2004). The latter involves the production of secreted antibiotic compounds that may move systemically throughout colonized plants as well as via induction of plant defenses (Guzman-Guzman et al., 2019).

For grapevines, several studies have tested *Trichoderma*, generally isolates of *Trichoderma atroviride* or *Trichoderma harzianum*, or isolates later often reclassified as *Trichoderma afroharzianum* (Chaverri et al., 2015), for control of different fungal trunk diseases, such as black foot, esca, *Eutypa* dieback, and nursery diseases (Fourie et al., 2001; Di Marco et al., 2004; John et al., 2005; Fourie and Halleen, 2006; Berlanas et al., 2018; Marraschi et al., 2019; Berbegal et al., 2020; Blundell and Eskalen, 2022). However, it should be noted that previous studies have been limited in the number of isolates tested and limited in the climates where said studies were performed. This likely results in a challenge in determining the suitability of various *Trichoderma* species to serve as effective controls in a particular vineyard. As such, efforts have been undertaken to isolate a greater variety of *Trichoderma* isolates specifically for tests in vineyards, from different climates, such as in Canada (Pollard-Flamand et al., 2022) and Italy (Urbez-Torres et al., 2020). This could provide better biological control agents, as the use of *Trichoderma* isolates from the same climate and host should presumably be better suited to limit pathogens (Pollard-Flamand et al., 2022; Chan et al., 2023) and could even discover novel species endemic to the region that are usable for effective biological control (Pollard-Flamand et al., 2022). Furthermore, the use of isolates within the same region collected could greatly simplify the registration process, as exploiting an organism as a biological control agent in its original region should pose no additional threat to non-target organisms, and therefore, such testing will not be necessary.

Therefore, this study was initiated to obtain new *Trichoderma* isolates for use as biological control agents in vineyards in the semi-arid climate of California. In addition to examining the ability of novel isolates to limit fungal trunk pathogens, their capacity to reduce the development of Pierce’s disease was also examined. Gained knowledge should be useful in determining whether the region-specific isolation of *Trichoderma* could be viable to generate new biological control agents to manage various grapevine diseases.

## 2 Materials and methods

### 2.1 Fungal isolations and culturing conditions

In the summer of 2020, leaf, stem, and root tissues from grapevines growing throughout central California were collected. Locations sampled included vineyards within and near Bakersfield, Delano, King City, Parlier, Reedley, San Luis Obispo, and Tooleville, CA, USA. This involved surface-sterilizing 1–2-cm segments of stems or roots, or small discs (1-cm diameter) of leaves, in 10% bleach for 1 minute, followed by two washes in sterile deionized water. The tissues were then placed onto sterile potato dextrose agar (PDA; Difco, Thermo-Fisher, Waltham, MA, USA). Emergent fungi were hyphal tip-cultured to obtain pure cultures. Cultures that had green sporulation were then separated, as these would most likely be *Trichoderma* species. Morphological assessments further confirmed the cultures that belonged to the *Trichoderma* genus following a dichotomous key (Samuels and Hebbar, 2016). This resulted in 30 potential *Trichoderma* isolates across all vineyards and tissues. Isolates similar in morphology and from the same sites and tissues were considered to be potentially the same isolate, and therefore, only one of these was tested further, with the others kept in glycerol freezer stocks. *Trichoderma* isolates, as well as pathogen isolates of *D. seriata*, *E. lata*, *N. parvum*, or *P. chlamydospora*, were maintained on PDA plates grown in the dark at 24 °C. Descriptions of these isolates are provided in **Table 1**.

**Table 1:** Fungal species analyzed in this study.

Fungal species	Isolate	Origin (CA, USA)^1^	Reference/initial source
*Diplodia seriata*	SBen831	San Benito Co.	Morales-Cruz et al., 2015
*Eutypa lata*	Napa209	Napa Co.	Travadon and Baumgartner, 2015
*Neofusicoccum parvum*	UCD646So	Sonoma Co.	Úrbez-Torres et al., 2006; Travadon et al., 2013; Czemmel et al., 2015; Galarneau et al., 2019
*Trichoderma asperellum*	TSI	Tulare Co.	Antrim et al., 2025
*Trichoderma saturnisporopsis*	RSI	Fresno Co.	Antrim et al., 2025
*Trichoderma* sp. DL1-3	DL1-3	Kern Co.	This paper
*Trichoderma harzianum*	KC1-1	Monterey Co.	This paper
*Trichoderma capillare*	KC2-2	Monterey Co.	This paper
*T. harzianum*	PAR3	Fresno Co.	Wallis et al., 2022a Gorman et al., 2023
*Trichoderma* sp. PAR10	PAR10	Fresno Co.	This paper
*T. capillare*	SLO1-1	San Luis Obispo Co.	Chen et al., 2014

^1^ All fungi were isolated in California, USA. The county of isolation is provided if known. All were isolated from *Vitis vinifera* except the isolate of *E. lata*, which was from *Prunus armeniaca*.

### 2.2 Preliminary culture antagonism assays and further *Trichoderma* identifications

Initial culture antagonism assays were conducted to screen and reduce the total number of *Trichoderma* isolates to a more manageable number for further assays. These consisted of plating a *Trichoderma* isolate approximately 2cm from the edge of a 100-mm PDA plate and then placing *D. seriata*, *E. lata*, or *N. parvum* approximately 2cm from the opposite end of the plate. Negative controls involved plating the pathogens by themselves. Four replicate plates of each *Trichoderma* isolate and pathogen combination were used. Radiuses in two directions of the pathogen colonies were measured at 3 days of growth. Percent inhibition was calculated as the difference between the mean radius of a pathogen colony co-plated with a *Trichoderma* isolate and the mean radius of a pathogen colony grown by itself, divided by the radiuses of the colonies on the co-plated experimental plates, and multiplied by 100%.

In addition to co-plating assays, the ability of compounds produced by six different *Trichoderma* isolates was assessed to reduce the colony growth of *D. seriata*, *E. lata*, or *N. parvum* on plates amended with spent media. The co-plating assays observed that the presence of a fungal pathogen increased the pigmented metabolite production of *Trichoderma* in the growth media. Thus, to acquire *Trichoderma* compounds, *N. parvum* was first grown in 500 mL of potato dextrose broth (PDB; Difco) in 1,000-mL Erlenmeyer flasks on a shaker (100 rpm) at 26 °C for 1 week. These colonies were then autoclaved to kill the *N. parvum* pathogen colonies, after which one of the six isolates of *Trichoderma* was added and grown for 1 week under similar conditions, with other media left sterile as a control. Following the week of *Trichoderma* isolate growth, the PDB cultures were filtered through 1-μm filters (Millipore Sigma, St. Louis, MO, USA) to obtain sterile spent media. Potato dextrose agar with half of the usual water added was autoclaved, and afterward, the sterile spent media were added to make up for the missing water prior to pouring the culture plates (resulting in a medium comprised of 50% of the original concentration of the spent broth). Colonies of *D. seriata*, *E. lata*, or *N. parvum* were then started in the middle of these plates, with growth measured as colony radiuses after 3 days of growth. Each treatment had 10 plates used. Percent inhibition was calculated similarly to the co-plating assays.

The six isolates with the most inhibitory capacity (i.e., DL1-3, KC1-1, KC2-2, PAR3, PAR10, and SLO1-1) were chosen for their ability to reduce infections in greenhouse and field assays. They were also sequenced to identify the isolates to species. DNA was extracted using the Quick-DNA Plant Kit (Zymo Research, Tustin, CA, USA), with DNA quantified by Invitrogen (Waltham, MA, USA) Qubit and quality-assessed using an Agilent (Santa Clara, CA, USA) TapeStation following the manufacturer’s protocols. The high-quality DNA was then sent for short-read sequencing using an Illumina (Torrance, CA, USA) NovaSeq 6000 via a commercial provider, Novogene (Durham, NC, USA), for DL1-3, KC1-1, PAR3, PAR10, and SLO1-1. Sequence reads were assembled using SPAdes ver. 3.14.0 (Bankevich et al., 2012), with quality of the assembly assessed using QUAST (Gurevich et al., 2013). For KC2-2, DNA was sequenced using the Oxford Nanopore (San Francisco, CA, USA) PromethION instrument (with R10.4.1 flow cells and v14 chemistry), with Flye ver. 2.9.1 (Kolmogorov et al., 2019) used for selecting high-quality reads, Medaka ver. 1.8.0 (Oxford Nanopore) for polishing, and Busco ver. 5.7.1 (Simao et al., 2015) for quality assessment. For all sequences, stand-alone BLAST+ (Camacho et al., 2009) was then used to identify the internal transcribed spacer (ITS) region, the RNA polymerase II (*rpb2*), and the translation elongation factor 1-α (*tef1*) genes. The iterative process described by Cai and Druzhinina (2021) was then used to identify *Trichoderma* to species. This involved first determining if the percent identity of the ITS region was greater than 76% between unknown isolates and at least one known *Trichoderma* species (Cai and Druzhinina, 2021). The ITS sequences were aligned by inserting those from these isolates into the Nexus file provided as **Supplementary Data Sheet** in Cai and Druzhinina (2021). Next, curated collections of closely related *Trichoderma* sp. for both the *rpb2* and *tef1* genes were obtained. These were used to determine if the percent identities would be over 99% for *rpb2* and over 97% for *tef1* when comparing the sequence from an unidentified isolate with that of known *Trichoderma* sp., in which case a match that met the criteria would identify the isolate’s species (Cai and Druzhinina, 2021). If not, phylogenetic trees would be examined to determine if an isolate is a putative novel species (Cai and Druzhinina, 2021). Alignment of these sequences is available in **Supplementary Data Sheet 1** and **2**. To further confirm species, colony morphology (with pictures available in **Supplementary Data Sheet 3**) and microscopy were performed to verify that the characteristics of colonies were consistent with the sequencing identifications (Samuels and Hebbar, 2016).

### 2.3 Greenhouse assays to assess the capacity of *Trichoderma* isolates to reduce pathogen infections

Grapevines used in all greenhouse experiments were Cabernet Sauvignon grafted onto 101–14 MG rootstocks and were kept in climate-controlled conditions (temperatures averaging 20 °C to 30 °C, humidity approximately 25%, and supplemental lighting set to maintain 14 hours of daylight).

Greenhouse experiments were replicated in full twice, once in 2023 and once in 2024. In 2023, only one isolate of each species was examined (i.e., DL1-3, KC1-1, PAR10, and SLO1-1). In 2024, all isolates from this study were used (i.e., DL1-3, KC1-1, KC2-2, PAR3, PAR10, and SLO1-1) as well as two additional promising isolates from California identified in another study: RSI (previously identified as *Trichoderma saturnisporopsis*) and TLI (previously identified as *Trichoderma asperellum*) (Antrim et al., 2025).

All treatments were placed in a completely randomized block experimental design, with two spatial blocks used per experiment. Treatments consisted of a first inoculation treatment of either a mock inoculation (on five plants in 2023 or 10 plants in 2024) or inoculation of one of the *Trichoderma* isolates (on five plants each during both years). Inoculations consisted of creating a 10-mm wound in the bark of the grapevine with a cork borer in the middle of the first internode of the scion after the scion-rootstock graft site. To this wound, a colonized 8-mm PDA plug (or uncolonized sterile plug for mock-inoculated controls) was applied, mycelium-side down, and wrapped in parafilm to allow colonization to occur.

Two weeks after the *Trichoderma* inoculations, pathogen inoculations occurred. This involved a mock inoculation, inoculations by the fungal pathogens *D. seriata* or *N. parvum* (performed similarly with colonized agar plugs that were performed with the *Trichoderma* inoculations), or inoculation by the bacteria *X. fastidiosa* ssp. *fastidiosa* strain SL (Hopkins, 2001). The fungal inoculations or mock controls were applied to the first internode after the scion-rootstock graft on a different branch than the *Trichoderma* sites or, when only one branch was available, applied to two internodes above the *Trichoderma* inoculation site.

Prior to inoculation, *X. fastidiosa* was maintained on periwinkle wilt media for 2 weeks in darkness at 24 °C. *X. fastidiosa* inoculations were performed via the pin-prick method. In brief, bacteria were harvested from culture plates, and a suspension of approximately 1 × 10^5^ CFUs/mL was made in sterile water. An 18-gauge needle was used to make five wound sites (on locations similar to where the fungal pathogens were applied as described above), and approximately 10 µL of bacterial suspension was applied to those wounds, allowing the bacteria to enter the branch via capillary action.

Six months after the pathogen treatments, disease symptoms were assessed by photographing all grapevines. Disease severity was rated on a 0–5 scale, similar to a commonly used scale for Pierce’s diseases (Wallis and Chen, 2012), with “0” meaning no symptoms or leaf damage, “4” meaning severe damage, and “5” meaning plant death. In addition to foliar damage and discoloration, this scale also incorporated relative decreases in new internode lengths and leaf size, as these could be symptoms of Pierce’s disease or fungal canker disease.

Following symptom assessment, all plants were destructively harvested to quantify infections. At the fungal inoculation sites, development of cankers was assessed using a ruler to measure both external cankers, and developing internal discoloration was reviewed after de-barking the infected internode segment. Approximately 1-cm segments in the apical and basal sides of the developing canker were collected, surface-sterilized in 10% bleach for 1 minute, washed in sterile water twice, and then plated on sterile PDA to confirm *Trichoderma* or pathogen fungal recovery. Confirmations were made via morphology observations. Isolate-treated vines that were negative for *Trichoderma* were removed from further analyses, as well as when non-*Trichoderma* controls were positive for *Trichoderma*. Likewise, mock-inoculated vines that failed to test positive for the subsequent pathogen infections were also removed from analyses.

For *X. fastidiosa*-inoculated grapevines, stem segments from the initial inoculation site were pulverized in liquid nitrogen, and DNA was extracted as described above. Real-time quantitative PCR was then performed using the primers and methods as described in Chen et al. (2005) using a Bio-Rad (Hercules, CA, USA) qPCR thermocycler and associated software.

### 2.4 Field efficacy of *Trichoderma* to control fungal pathogens in grapevine spurs

The six *Trichoderma* isolates selected from this study (DL1-3, KC1-1, KC2-2, SLO1-1, PAR3, and PAR10), as well as the two from a previous study (RSI and TLI), were assessed for their ability to prevent or remove naturally occurring fungal canker pathogens in an over 10-year-old Cabernet Sauvignon vineyard. The experiment was performed in both 2023 and 2024.

A total of 24 vines in 2023 and 12 vines in 2024 received one of the following treatments on six (for 2023) or three (for 2024) freshly pruned spurs per vine in May, arranged in a completely randomized block design with three spatial blocks: inoculation with one of the eight *Trichoderma* isolates (at a concentration of 100,000 spores/mL), mock inoculation with sterile water, or treatment with a fungicide (Topsin M, or thiophanate-methyl, at 1.8 g/L). Spore suspensions, water, or fungicide was applied using a micropipette with enough liquid to completely cover a cut spur, approximately 0.5 to 1 mL of solution per spur.

Six months after treatment, spurs were harvested. For each spur, two horizontal segments (approximately 10mm thick) were collected. The apical segment was surface-sterilized in 10% bleach for 1 minute, washed twice in sterile water, and plated on PDA. Plates were observed at 5 days, with the emergent fungi noted and identified via morphology. Pictures were taken to assist in data collection.

### 2.5 Statistical analyses

JMP version 17 (SAS Institute, Cary, NC, USA) was used for all statistical analyses, with α = 0.05. For analyses that required normal distributions, normality of the data was assessed using a quantile–quantile (QQ) plot and the Shapiro–Wilk test. Differences between growth inhibition, external canker lengths, internal discoloration, and pathogen recoveries or *Trichoderma* recoveries between treatments were determined using analysis of variance (ANOVA) tests with follow-up least significant difference (LSD) mean separation tests. Differences in Pierce’s disease symptoms and *X. fastidiosa* titers were determined by non-parametric Kruskal–Wallis tests with follow-up Wilcoxon pairwise comparisons, as the data were non-continuous or did not meet normality assumptions.

## 3 Results

### 3.1 *Trichoderma* isolate species identification

Every isolate that was sequenced had a percent similarity index greater than 76% with multiple existing *Trichoderma* species, and therefore, all were concluded to be members of the *Trichoderma* genus.

DL1–3 had the greatest similarity index value of *rpb2* of 97.49% with *Trichoderma guizhouense* and a similarity index of *tef1* intron 4 of 95.95% with *Trichoderma rifaii*. The findings were precise, accurate, and unambiguous that a putative new species had non-concordant phylogenies of *rpb2* and *tef1*. DL1–3 was therefore considered a putative novel species, *Trichoderma* sp. DL1-3. Because of the potential as a biological control agent, we propose a provisional name of *Trichoderma kernensis*, after Kern County, California, USA, where it is from, although a great morphological description is needed.

KC1–1 and PAR3 were 100% similar for the *rpb2* and *tef1* sequences. Both had the greatest similarity of *rpb2* of 99.30% to *T. harzianum* and similarity of *tef1* intron 4 of 100% with *T. harzianum*. The identification was precise, accurate, and unambiguous that KC1–1 and PAR3 were isolates of *T. harzianum*.

KC2–2 and SLO1–1 had identical *rpb2* and *tef1* sequences. They had the greatest similarity of *rpb2* of 99.58% with *Trichoderma capillare* and similarity of *tef1* intron 4 of 99.28% with *T. capillare*. Therefore, the results were precise, accurate, and unambiguous that KC2–2 and SLO1–1 were identified as *T. capillare*.

PAR10 had the greatest similarity of *rpb2* of 96.94% with three species (*T. guizhouense*, *Trichoderma pyramidale*, and *Trichoderma simmonsii*) and similarity of *tef1* intron 4 of 90.44% with *T. pyramidale*. These results were precise, accurate, and unambiguous and suggested that PAR10 is a new species, related to *Trichoderma pyramidale*, and described as *Trichoderma* sp. PAR10. Due to the potential importance in biological control, we propose the name of *Trichoderma parlierensis*, after Parlier, CA, USA, the town from which it was isolated, although a greater morphological description is needed.

### 3.2 Ability of *Trichoderma* isolates to inhibit pathogen growth on culture plates

**Figure 1:**
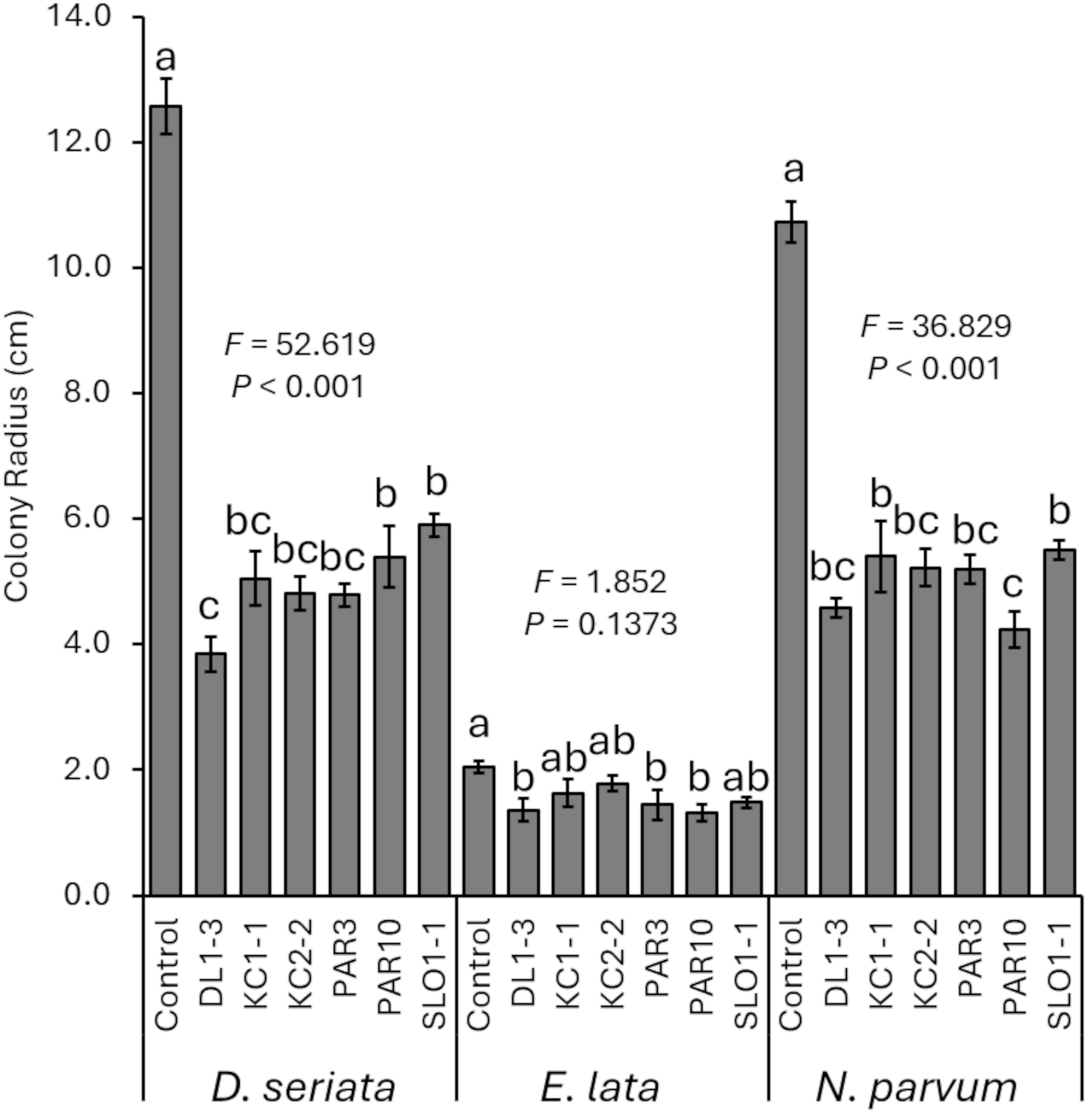
Mean radius of pathogen colonies when co-plated with one of the six *Trichoderma* isolates. Error bars represent standard errors. ANOVA statistics are provided for each fungal pathogen, and different letters represent significant differences by LSD tests. LSD, least significant difference.

All selected isolates successfully reduced pathogen growth (or had a greater mean inhibition) when co-plated with pathogens *D. seriata* (*F*_6, 26_ = 52.619; *p* < 0.001) and *N. parvum* (*F*_6, 26_ = 36.829; *p* < 0.001), but not significantly so for *E. lata* (*F*_6, 26_ = 1.852; *p* = 0.137) (**Figure 1**). However, follow-up LSD tests did reveal that when *E. lata* colonies were co-plated with DL1-3, PAR3, and PAR10, the sizes were significantly smaller according to LSD tests than when *E. lata* was not co-plated. For *D. seriata*, colonies co-plated with DL1–3 were also significantly smaller than colonies co-plated with either PAR10 or SLO1-1. For *N. parvum*, colonies co-plated with either KC1–1 or SLO1–1 were significantly smaller than colonies co-plated with PAR10.

**Figure 2:**
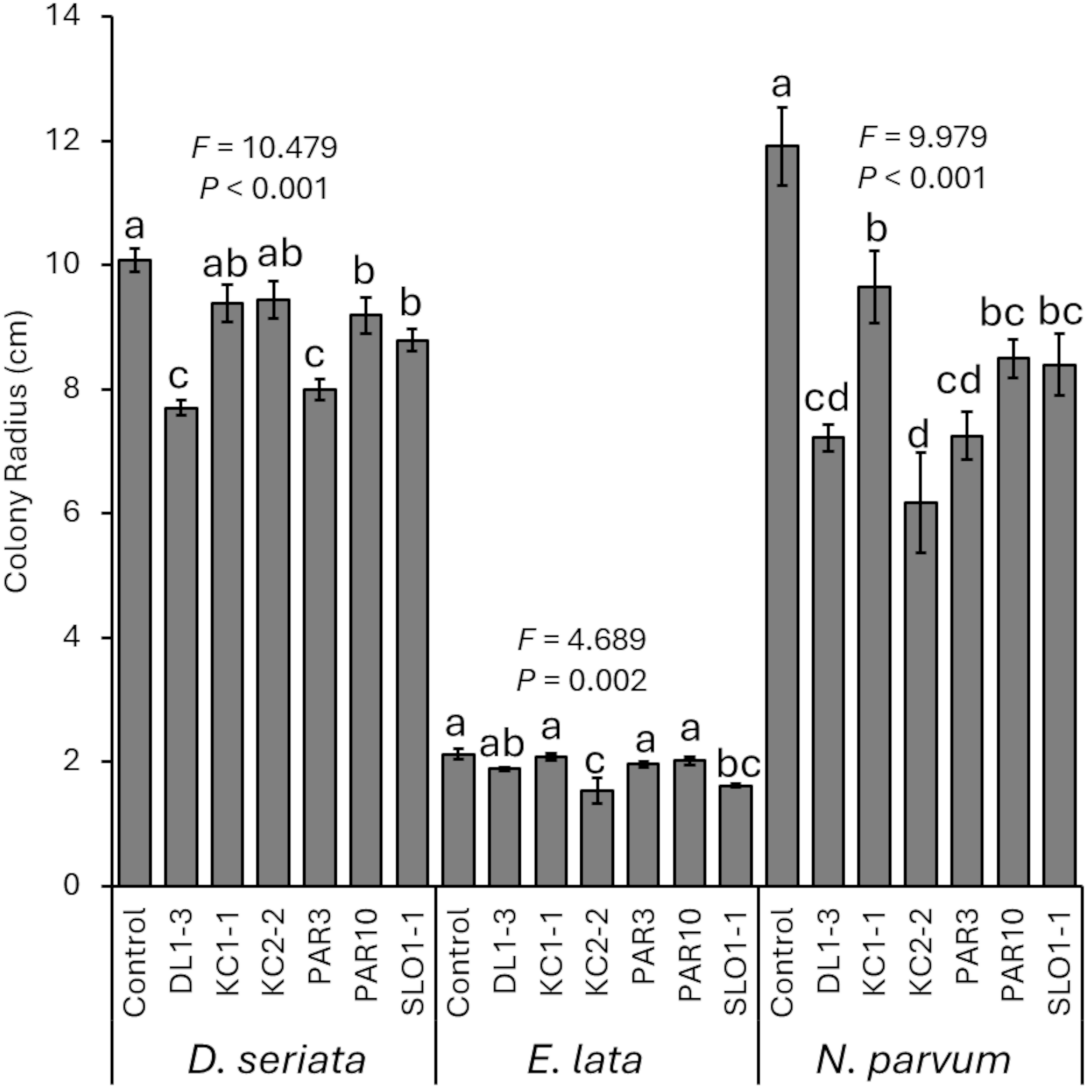
Mean percent reduction of pathogen colonies when grown on spent media from one of the six *Trichoderma* isolates. Error bars represent standard errors. ANOVA statistics are provided for each fungal pathogen, and different letters represent significant differences by LSD tests. LSD, least significant difference.

When grown on PDA plates amended with media that previously had the *Trichoderma* isolates grown (“spent media”), *D. seriata* (*F*_6, 28_ = 10.479; *p* < 0.001), *E. lata* (*F*_6, 28_ = 4.689; *p* = 0.002), and *N. parvum* (*F*_6, 25_ = 9.979; *p* < 0.001) had reduced colony growth (greater inhibition of growth) compared with controls (**Figure 2**). For *D. seriata*, LSD tests revealed significant differences in colonies grown on control agar compared with those grown on spent media from all isolates except KC1–1 and KC2-2. In addition, colonies grown on spent media from DL1–3 or PAR3 were smaller than those grown on spent media with the other isolates. For *E. lata*, LSD tests revealed that colonies grown on control media were significantly larger than those grown on spent media from KC2–2 or SLO1-1. The use of spent media from SLO1–1 also reduced *E. lata* colonies more than spent media from all others except DL1–3 and KC2-2, and spent media from KC2–2 reduced growth more than spent media from all other isolates except SLO1-1. Lastly, *N. parvum* colonies grown on control plates were significantly larger than those on colonies grown on spent media plates. *N. parvum* colonies grown on KC1–1 spent media were also significantly larger than those grown on DL1-3, KC2-2, or PAR3 spent media.

### 3.3 Capacity of *Trichoderma* stem inoculations to reduce disease

For 2023, the ability of one isolate each of *T. harzianum* (KC1-1), *T. capillare* (SLO1-1), and the two putative new species (*Trichoderma* sp. DL1–3 and *Trichoderma* sp. PAR10) was assessed to reduce fungal canker pathogen diseases or Pierce’s diseases. For 2024, the additional isolates of *T. harzianum* (PAR3) and *T. capillare* (KC2-2), as well as the two isolates from Antrim et al. (2025) (*T. asperellum* isolate TLI and *T. saturnisporopsis* isolate RSI), were added to the 2023 tested isolates in the experimental trial.

**Figure 3:**
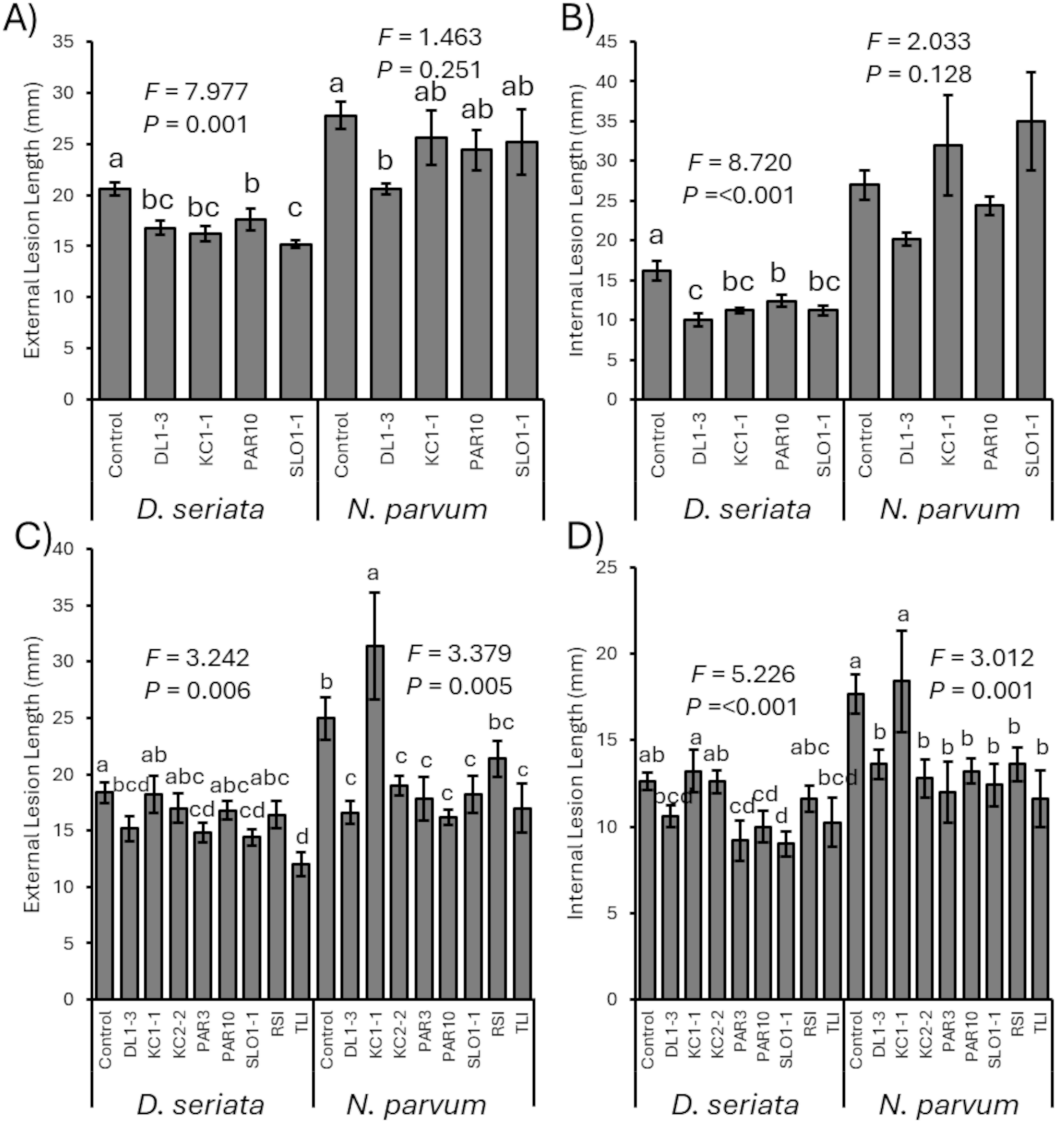
**(A)** Mean developing external canker length of *Diplodia seriata* or *Neofusicoccum parvum* infections in 2023 trials. **(B)** Mean internal discoloration caused by *D*. *seriata* or *N. parvum* infections in 2023 trials. **(C)** Mean developing external canker length of *D*. *seriata* or *N. parvum* infections in 2024 trials. **(D)** Mean internal discoloration caused by *D*. *seriata* or *N. parvum* infections in 2024 trials. Error bars represent standard errors. ANOVA statistics are provided for each fungal pathogen, and different letters represent significant differences by LSD tests. LSD, least significant difference.

In 2023, prior inoculation of any of the four isolates (DL1-3, KC1-1, PAR10, or SLO1-1) was able to generally significantly reduce subsequent *D. seriata* cankers compared to controls, whether measured as external cankers (*F*_4, 24_ = 7.977; *p* = 0.001) (**Figure 3A**) or interior discoloration (*F*_4, 24_ = 8.720; *p* < 0.001) (**Figure 3B**). In addition, vines treated initially with DL1–3 had smaller subsequent *D. seriata* cankers developed than those treated initially with PAR10.

Also in 2023, vines inoculated initially with any of the *Trichoderma* isolates did not have significant differences in canker development of subsequent *N. parvum* inoculations, measured as either external cankers (*F*_4, 24_ = 1.463; *p* = 0.251) (**Figure 3A**) or internal discoloration (*F*_4, 24_ = 2.033; *p* = 0.128) (**Figure 3B**). However, LSD tests did suggest that prior inoculation with isolate DL1–3 had reduced *N. parvum* external cankers compared with control vines that did not have *Trichoderma* inoculation.

For the 2024 experimental trials, vines that were not inoculated with a *Trichoderma* isolate had greater *D. seriata* external lesions than those inoculated with isolates DL1-3, PAR3, SLO1-1, and TLI (*F*_8, 40_ = 3.242; *p* = 0.006) (**Figure 3C**), and control vines had greater internal discoloration than vines inoculated with PAR3, PAR10, and SLO1-1 (*F*_8_, _40_ = 5.226; *p* < 0.001) (**Figure 3D**). Vines inoculated with KC1–1 had more *D. seriata* canker growth and internal discoloration than vines inoculated with other isolates. For *N. parvum*, both mock-inoculated and KC1-1-inoculated vines had greater lesion lengths (*F*_8, 40_ = 3.379; *p* = 0.005) (**Figure 3C**) and internal discoloration (*F*_8, 40_ = 3.012; *p* = 0.001) (**Figure 3D**) than those inoculated with any of the *Trichoderma* isolates except KC1-1 (albeit mock-inoculated and RSI-treated vines did not have different external lesions).

**Figure 4:**
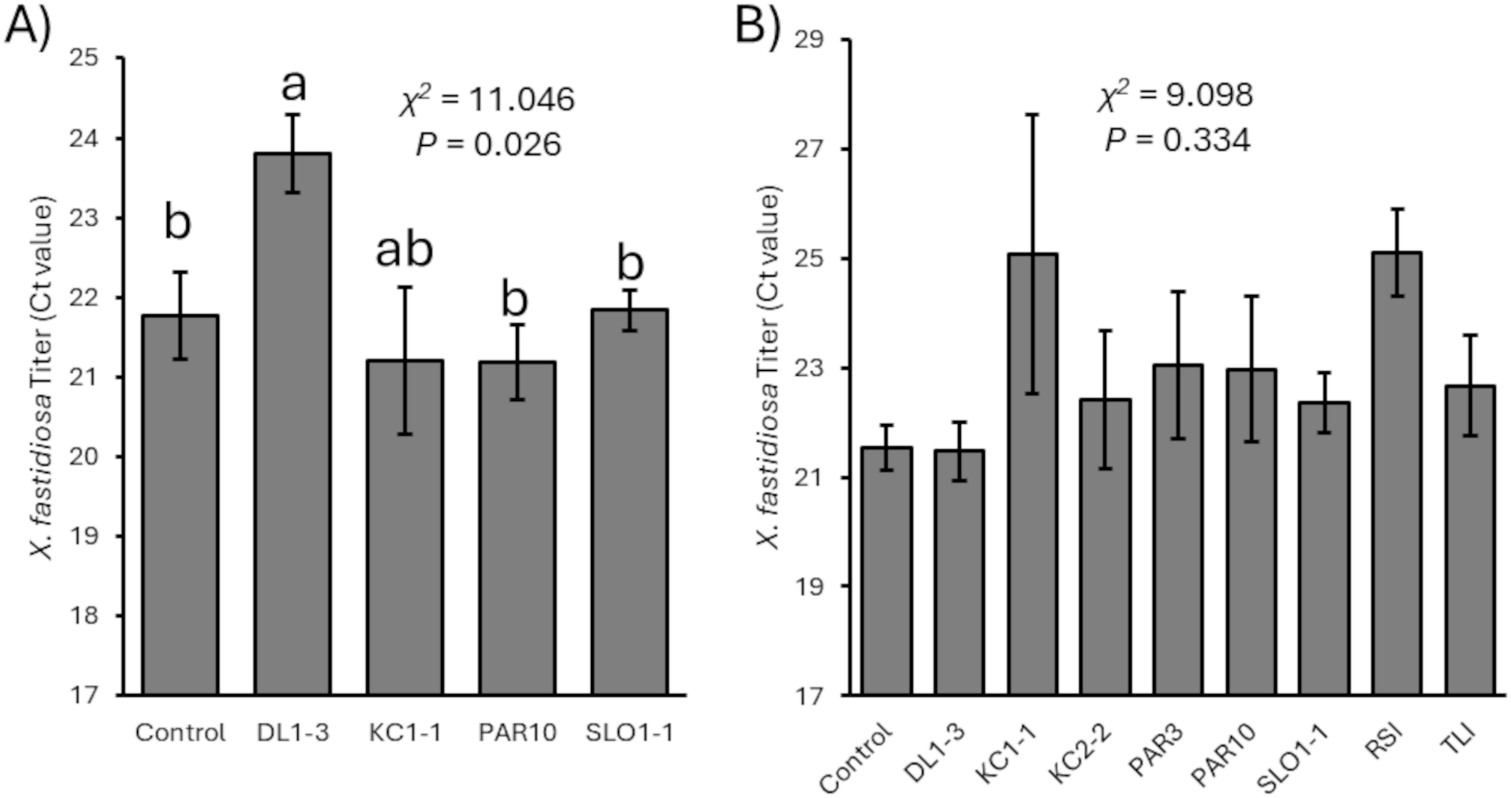
Mean Ct values related to *Xylella fastidiosa* titers (with greater Ct values representing lower titers) for **(A)** 2023 and **(B)** 2024 vines that were mock-inoculated or inoculated with a *Trichoderma* isolate. Error bars represent standard errors. Mann–Whitney U test statistics are provided, and different letters represent significant differences by Wilcoxon non-parametric pairwise tests. There were no significant differences in 2024 vines.

In both 2023 and 2024, prior treatment with the *Trichoderma* isolates did not alter the development of Pierce’s disease symptoms that followed *X. fastidiosa* inoculation when compared to controls (for 2023, χ^2^ = 2.626, *p* = 0.6222, N=25; for 2024, χ^2^ = 5.855, *p* = 0.663, N=50). Nevertheless, in 2023, measurements of *X. fastidiosa* titers by qPCR suggested significant differences in Ct values (with lower Ct values representing greater bacterial titers) between vines previously inoculated with isolate DL1–3 and those not previously inoculated or inoculated with isolate SLO1–1 or PAR10 (χ^2^ = 11.046, *p* = 0.026, N=24) (**Figure 4A**). However, no significant differences were observed (χ^2^ = 9.098, *p* = 0.334, N=50) in *X. fastidiosa* titers in the 2024 experimental trial (**Figure 4B**).

### 3.4 Capacity of *Trichoderma* isolates to prevent colonization of cut spurs

To assess the ability of the six *Trichoderma* isolates from this study and the two other promising isolates (Antrim et al., 2025), freshly cut spurs were inoculated, treated with fungicide, or mock-inoculated with water in May 2023 or 2024. Over 6 months later, the spurs were collected and assessed for the recovery of fungal pathogens or *Trichoderma* isolates. Note that negative controls may have already been infected with pre-existing fungal pathogens or *Trichoderma*.

**Figure 5:**
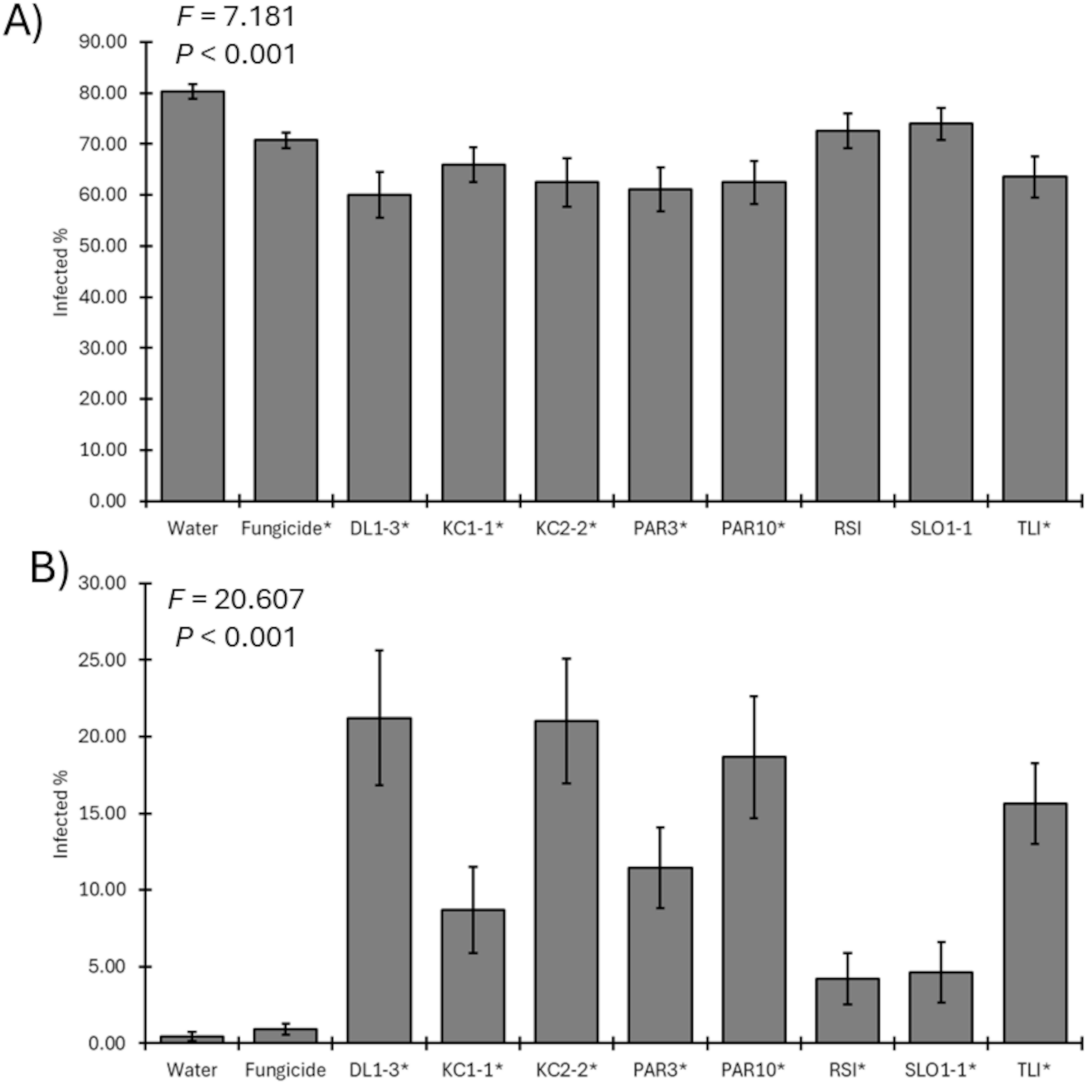
**(A)** Mean percent recovery of fungal pathogens in plants treated by mock inoculation, fungicide, or inoculation by a *Trichoderma* isolate for 2023 trial. ANOVA statistics are provided, and an asterisk represents isolates where recovery was lower than that of water-only negative controls. **(B)** Mean recovery percent of a *Trichoderma* isolate in plants treated by mock inoculation, fungicide, or isolate inoculation for 2023 trial. ANOVA statistics are provided, and an asterisk represents isolates where recovery was greater than that of water-only negative controls. Error bars represent standard errors.

In 2023, six of the eight *Trichoderma* isolates and the fungicide significantly reduced the recovery of the pathogen compared to the mock-inoculated control (*F*_9, 1113_ = 7.181; *p* < 0.001) (**Figure 5A**). Only isolates RSI and SLO1–1 did not result in reduced pathogen recoveries from the spurs compared with the water-only negative controls. Furthermore, all *Trichoderma* isolates were recovered in greater numbers in inoculated spurs rather than fungicide-treated spurs or controls over 6 months after application (*F*_9, 1113_ = 20.607; *p* < 0.001) (**Figure 5B**).

**Figure 6:**
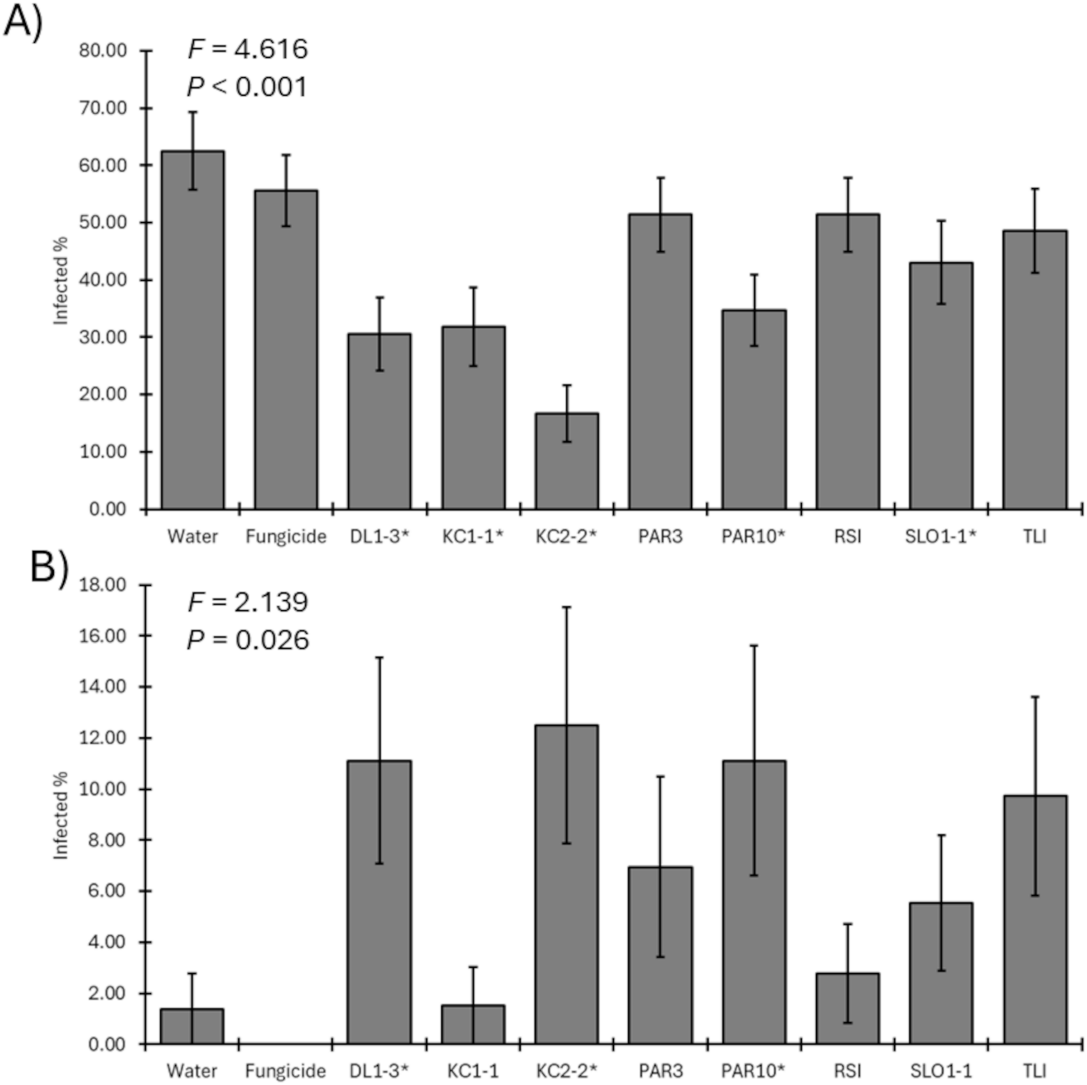
**(A)** Mean percent recovery of fungal pathogens in plants treated by mock inoculation, fungicide, or inoculation by a *Trichoderma* isolate for 2024 trial. ANOVA statistics are provided, and an asterisk represents isolates where recovery was lower than that of water-only negative controls. **(B)** Mean recovery percent of a *Trichoderma* isolate in plants treated by mock inoculation, fungicide, or isolate inoculation for 2024 trial. ANOVA statistics are provided, and an asterisk represents isolates where recovery was greater than that of water-only negative controls. Error bars represent standard errors.

For the 2024 field trial, five of the eight *Trichoderma* isolates significantly reduced the recovery of the pathogen compared to the mock-inoculated control (*F*_9, 356_ = 4.616; *p* < 0.001) (**Figure 6A**). Only isolates PAR3, RSI, and TLI did not result in reduced pathogen recoveries from the spurs compared with the water-only negative controls. Unlike 2023, the percent recovery of *Trichoderma* from treated spurs was only significantly greater for DL1-3, KC2-2, and PAR10 than *Trichoderma* recoveries from water-only negative controls (*F*_9, 356_ = 2.139; *p* = 0.026) (**Figure 6B**).

## 4 Discussion

These results observed the promise of a couple to several novel *Trichoderma* sp. isolates to outcompete or predate fungal pathogens to provide health benefits to grapevine hosts. This was demonstrated by observing direct predation via co-plating assays, the capacity of *Trichoderma* metabolites to inhibit pathogen growth, the reduction of developing pathogen lesions or titers in greenhouse experiments, or reduced pathogen recovery combined with *Trichoderma* recovery in treated spurs in vineyard studies. However, some inconsistencies were observed between years, implying the role of fluctuating weather conditions that may have influenced the results. For instance, it could be hypothesized that warmer, drier weather likely reduced the recovery of specific *Trichoderma* isolates and made them overall less likely to impair the targeted fungal pathogens. Furthermore, confirmation by sequencing of the recovered fungi would have been desirable and preferred in future follow-up studies, but could not be performed due to a large number of analyzed spurs (over 2,000 in 2023 and over 500 in 2024). Regardless, due to the sample size and previous studies that did sequence to confirm recoveries with great accuracy of over 95% (Wallis et al., 2022b; Sinclair et al., 2025), the statistical conclusions reached from this study would likely be similar.

Regardless, the objective of this study was to identify novel isolates of *Trichoderma* collected from grapevine tissues for re-application to grapevines to limit pathogen development in the hot, dry climate found in central California. To this end, promising isolates were identified and determined to be mostly in the *T. harzianum* complex, as well as *T. capillare*, *T. asperellum*, and *T. saturnisporopsis*. This was similar to studies by Pollard-Flamand et al. (2022) and Urbez-Torres et al. (2020), albeit these isolates were extracted directly from plant tissues in a different geographic location.

Regarding the differences in the isolates, *T. harzianum* and, to some degree, *T. asperellum* appeared to function differently than *T. capillare* SLO1-1. The former isolates appeared to aggressively predate the pathogens, whether by overrunning the plates in co-plating assays or perhaps overrunning spurs to remove and prevent pathogen establishment at those sites. By contrast, *T. capillare* SLO1–1 produced a more visibly pigmented compound in the spent media assays, which appeared to dramatically reduce pathogen growth. Likewise, systemic effects when applied to stem wounds appeared quite effective at reducing lesion sizes. However, SLO1–1 was not as readily recoverable in the spur experiments, nor was it able to remove pathogens from spurs as effectively.

Regarding *X. fastidiosa* and resultant Pierce’s disease, only one isolate (DL1-3) statistically reduced titers, implying a likely systemic effect to reduce the bacterial infections. It was possible that DL1–3 had produced its own compounds to affect *X. fastidiosa* directly, was able to induce changes in grapevine host physiology to alter the infection process, or some combination of both. Further research is warranted to explore what may be the cause of the observations.

In conclusion, these results identified different *Trichoderma* isolates that were observed to directly reduce pathogen growth and infection success and also had the capacity to survive in grapevine tissues throughout an entire growing season. Further studies will be needed to develop these further into potential products, and an improved understanding of isolate genomics and metabolomics will allow further conclusions on how they function to be reached.

## Data Availability

The datasets presented in this study can be found in online repositories. The names of the repository/repositories and accession number(s) can be found in the article/**Supplementary Material**.

## Supplementary material

Supplementary Data Sheet 1 File in the fasta format of the used *rpb2* sequences to perform alignments and allow the putative determination of collected *Trichoderma* strains for species identification purposes.

Supplementary Data Sheet 2 File in the fasta format of the used *rpb2* sequences to perform alignments and allow the putative determination of collected *Trichoderma* strains for species identification purposes.

Supplementary Data Sheet 3 Representative photographs of culture of each *Trichoderma* strain grown on potato dextrose agar (PDA) (left) or Spezieller Nahrstoffarmer agar (SNA) (right) after 1 week at 24 °C in darkness.
