# Supplementary figures and images for: Assessment of the potential of novel Californian grapevine Trichoderma isolates to reduce colonization of fungal trunk canker pathogens and Xylella fastidiosa
Source: Front Plant Sci. 2025 Sep 9;16:1609693. doi: 10.3389/fpls.2025.1609693 (PMC12454315; doi:10.3389/fpls.2025.1609693)

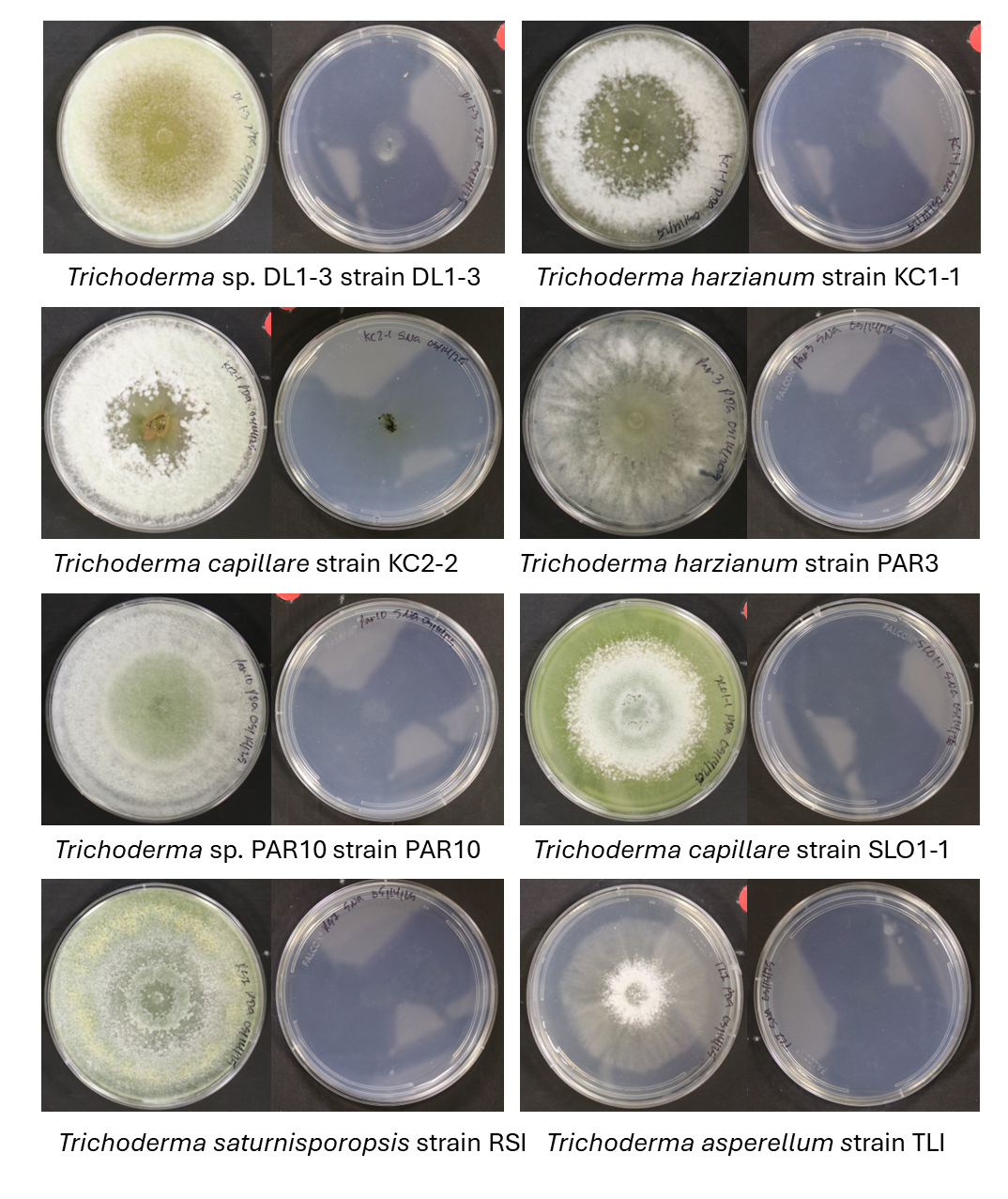

Supplement: Supplementary Data Sheet 1 — File in the fasta format of the used rpb2 sequences to perform alignments and allow the putative determination of collected Trichoderma strains for species identification purposes. [file Image1.tif]
